# Supplementary material for: Anti-Hyperalgesic Efficacy of Acetyl L-Carnitine (ALCAR) Against Visceral Pain Induced by Colitis: Involvement of Glia in the Enteric and Central Nervous System
Source: Int J Mol Sci. 2023 Oct 2;24(19):14841. doi: 10.3390/ijms241914841 (PMC10573187; doi:10.3390/ijms241914841)
Supplement: Supplementary file 1 [file ijms-24-14841-s001.zip › ijms-2476728-supplementary.pdf]

Supplementary Material

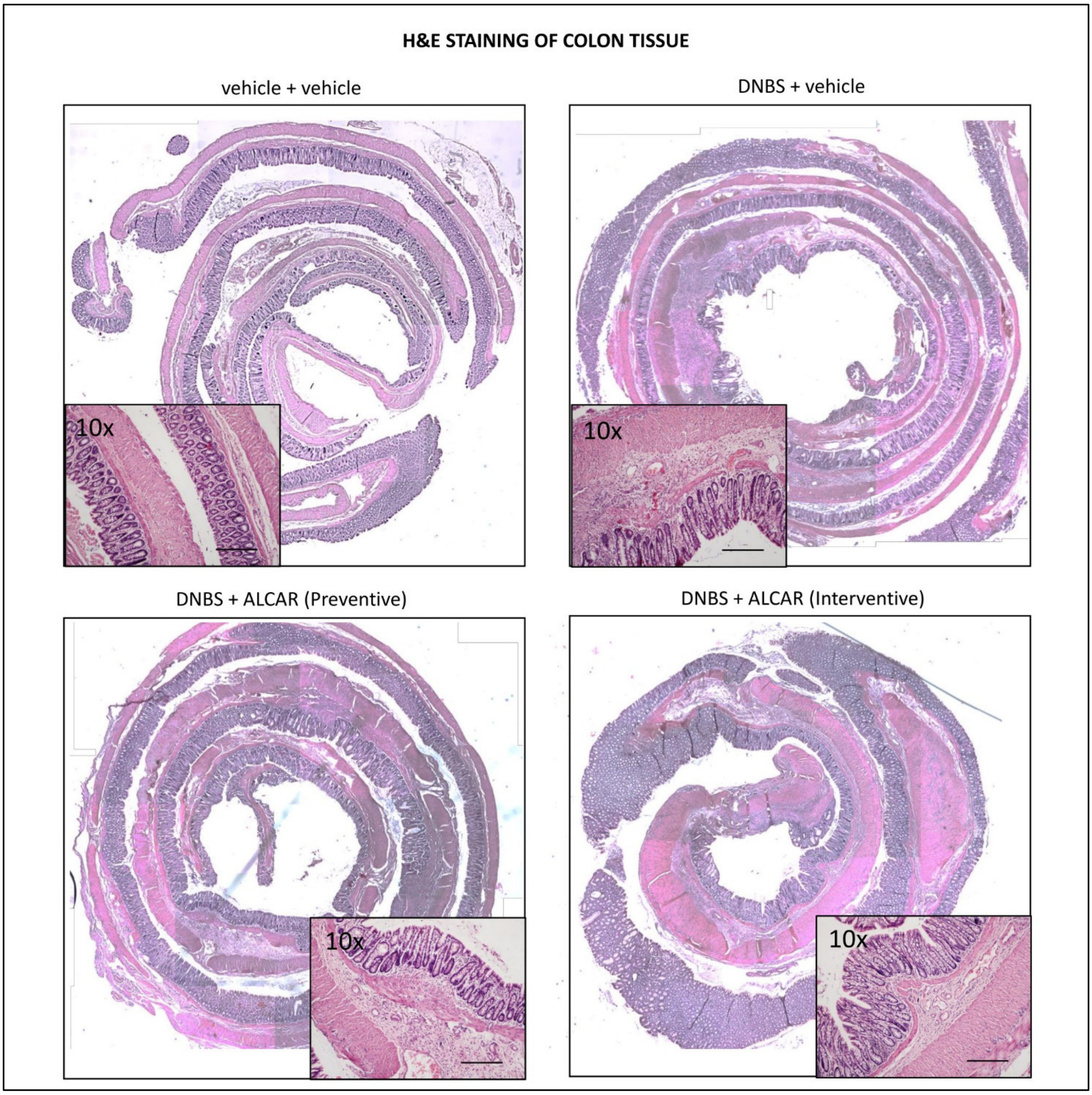

Figure S1. Representative images of H&E-stained colon of each experimental group. ALCAR (100 mg kg^{-1} s.c.) was administered twice daily in the DNBS-treated animals, according to the preventive and the interventive protocol, respectively; then tissues were collected on Day 16. The figure shows the H&E-stained “Swiss Role” of the colon where the images (Original magnification: 10×; insert) used for the analysis of colon damage were taken from.

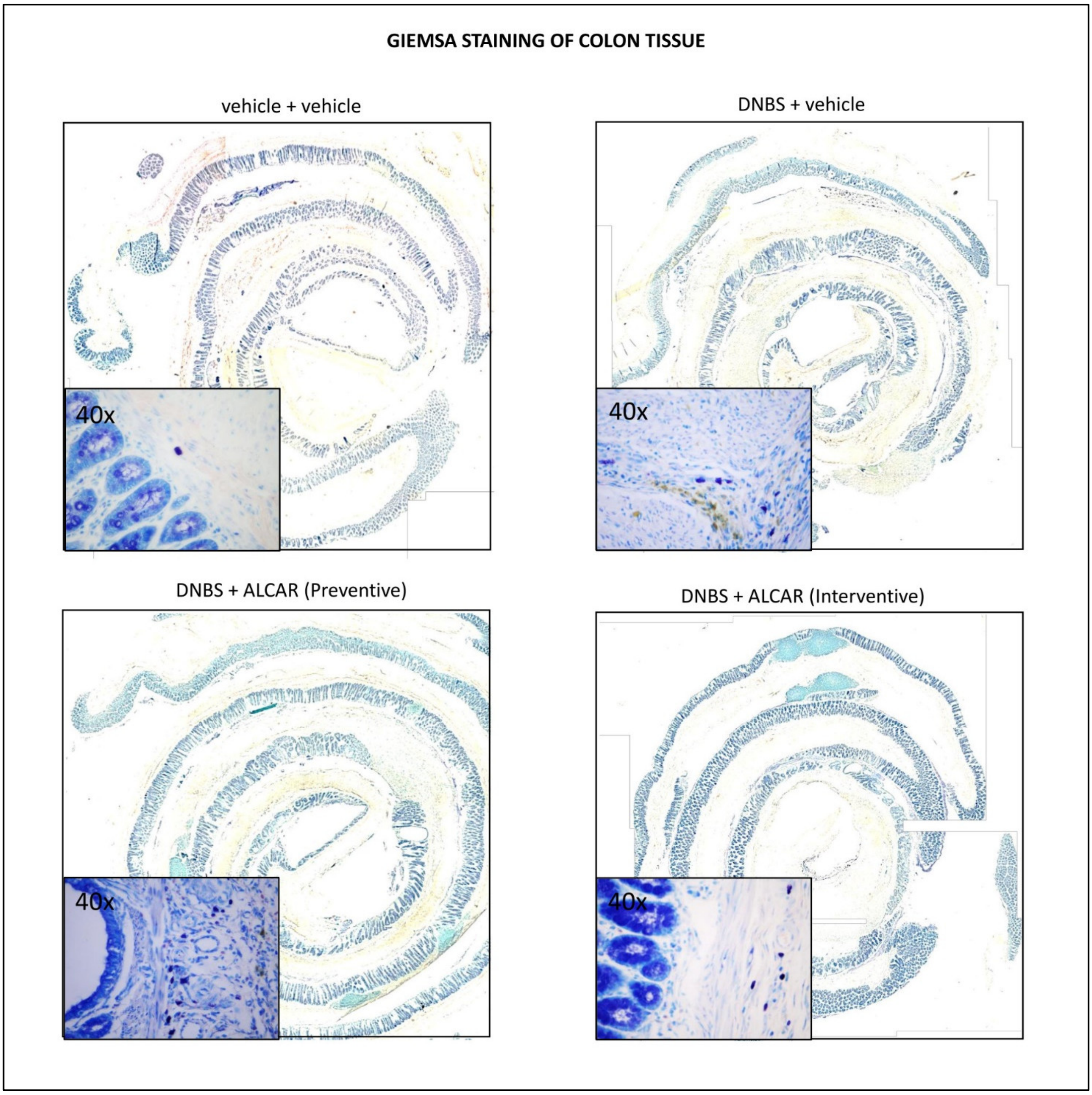

Figure S2. Representative images of GIEMSA-stained colon of each experimental group. ALCAR (100 mg kg^{-1} s.c.) was administered twice daily in the DNBS-treated animals, according to the preventive and the interventive protocol, respectively; then tissues were collected on Day 16. The figure shows the GIEMSA-stained "Swiss Role" of the colon where the images (Original magnification: 10×; insert) used for the analysis of mast cell and eosinophils density were taken from.

.

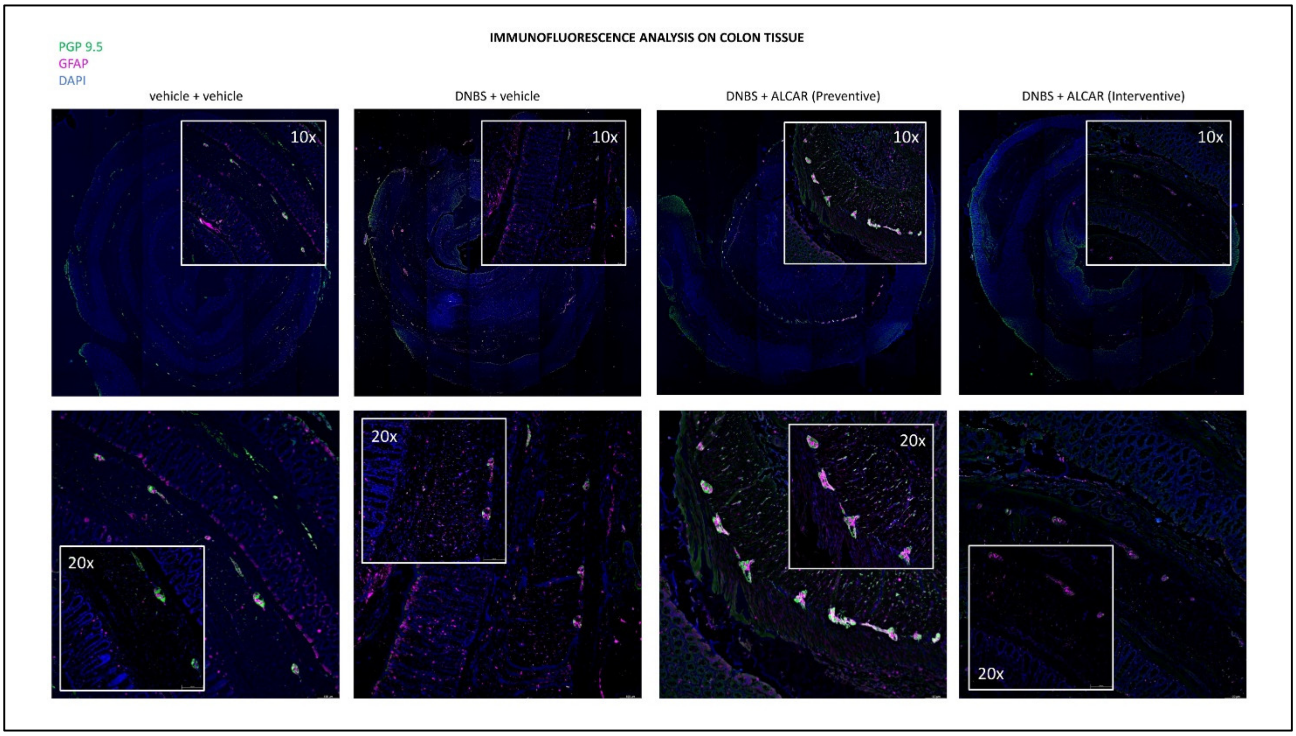

Figure S3. Representative images of PGP9.5 and GFAP expression in the colon of each experimental group. ALCAR (100 mg kg^{-1} s.c.) was administered twice daily in the DNBS-treated animals, according to the preventive and the interventive protocol, respectively; then tissues were collected on Day 16. The figure shows the overview of the "Swiss Role" of colon and the area (Original magnification: 10×; insert above) where the immunofluorescence images (Original magnification: 20×; below) used for the analysis of PGP9.5 and GFAP expression were taken from. PGP9.5 (green), GFAP (purple) and DAPI (blue; nuclei marker).

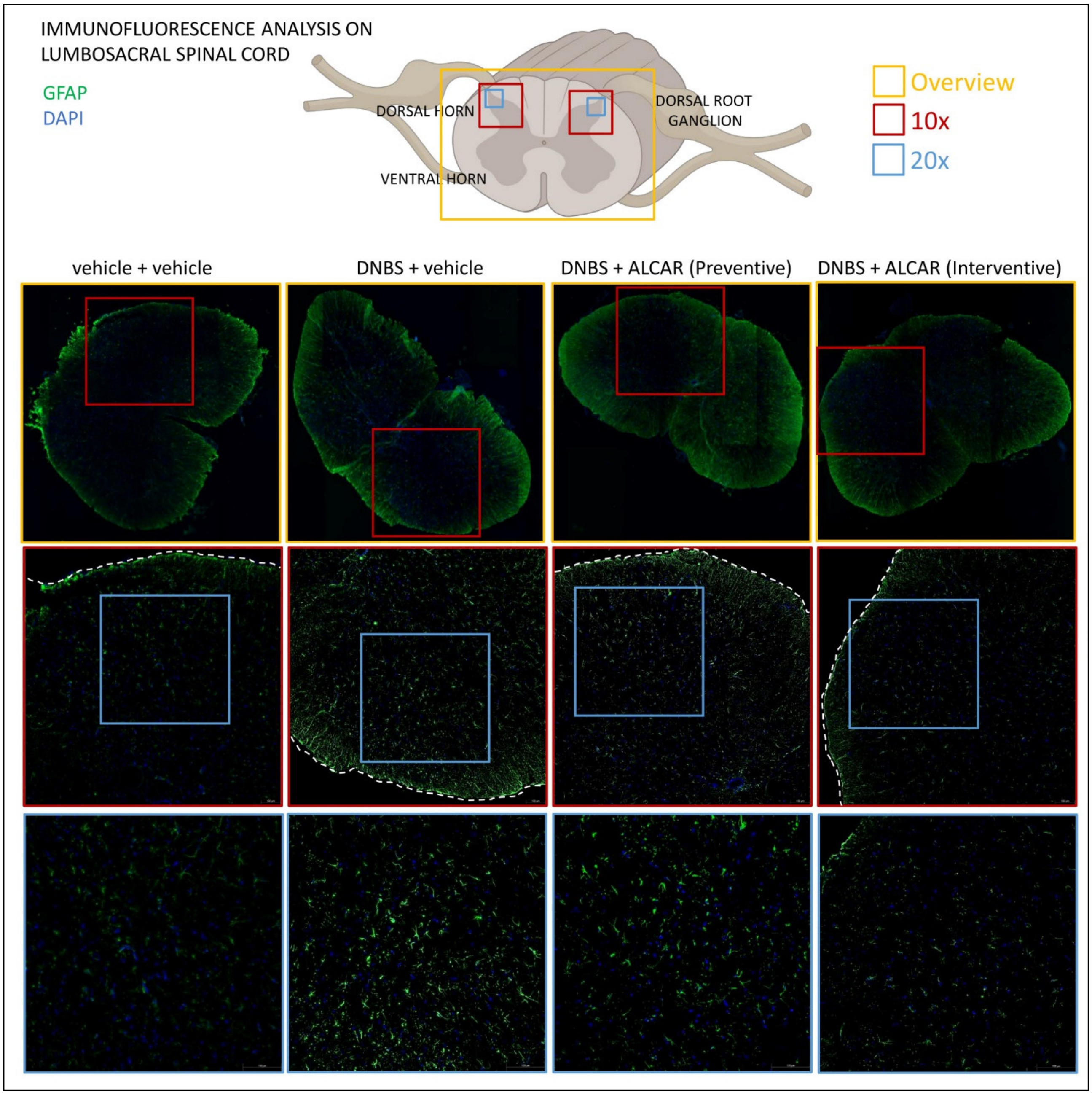

Figure S4. Representative images of GFAP expression in the dorsal horn of the spinal cord of each experimental group. ALCAR (100 mg kg^{-1} s.c.) was administered twice daily in the DNBS-treated animals, according to the preventive and the interventive protocol, respectively; then tissues were collected on Day 16. The figure shows the overview (above) of the spinal cord and the area of the dorsal horn of the spinal cord (Original magnification: 10×; middle) where the immunofluorescence images (Original magnification: 20×; below) used for the analysis of GFAP expression were taken from. GFAP (green) and DAPI (blue; nuclei marker).

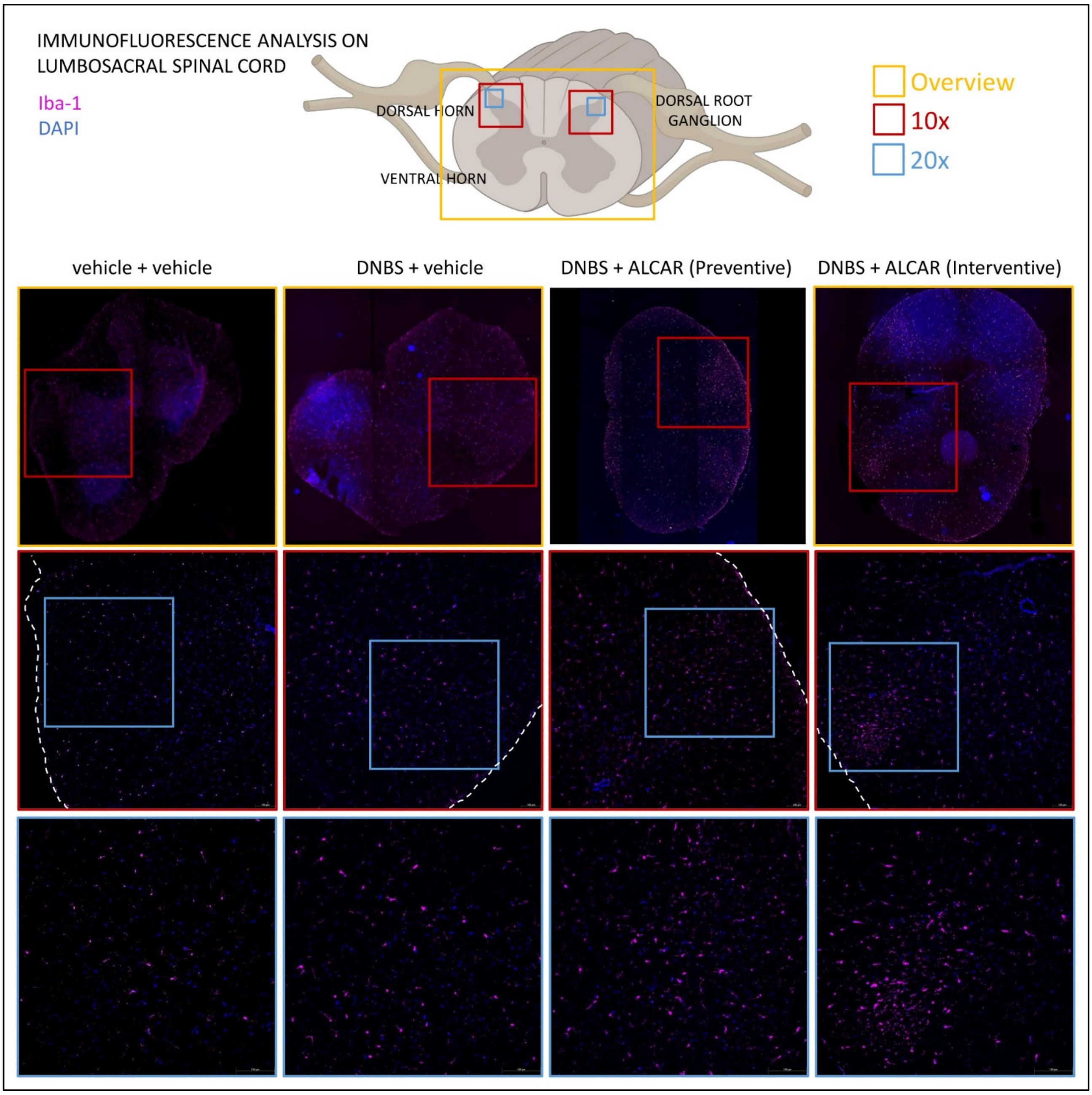

Figure S5. Representative images of Iba-1 expression in the dorsal horn of the spinal cord of each experimental group. ALCAR (100 mg kg^{-1} s.c.) was administered twice daily in the DNBS-treated animals, according to the preventive and the interventive protocol, respectively; then tissues were collected on Day 16. The figure shows the overview (above) of the spinal cord and the area of the dorsal horn of the spinal cord (Original magnification: 10×; middle) where the immunofluorescence images (Original magnification: 20×; below) used for the analysis of Iba-1 expression were taken from. Iba-1 (purple) and DAPI (blue; nuclei marker).
